# Supplementary material for: Mapping lesion-specific response and progression dynamics and inter-organ variability in metastatic colorectal cancer
Source: Nat Commun. 2023 Jan 26;14:417. doi: 10.1038/s41467-023-36121-y (PMC9876906; doi:10.1038/s41467-023-36121-y)
Supplement: Supplementary file 1 — Supplementary Information [file 41467_2023_36121_MOESM1_ESM.pdf]

Supplementary Information

Mapping Lesion-Specific Response and Progression Dynamics and Inter-Organ Variability in Metastatic Colorectal Cancer

Jiawei Zhou[1], Amber Cipriani[1,2], Yutong Liu[3], Gang Fang[4], Quefeng Li[3], Yanguang Cao[1,5*]

[1]Division of Pharmacotherapy and Experimental Therapeutics, School of Pharmacy, University of North Carolina at Chapel Hill, NC 27599, USA; [2]UNC Health Medical Center, Department of Pharmacy, Chapel Hill, NC 27514; [3]School of Public Health, University of North Carolina at Chapel Hill, NC 27599, USA; [4]Division of Pharmaceutical Outcomes and Policy, School of Pharmacy, University of North Carolina at Chapel Hill, NC 27599, USA; [5]Lineberger Comprehensive Cancer Center, School of Medicine, University of North Carolina at Chapel Hill, Chapel Hill, NC 27599, USA

Corresponding author:

Yanguang Cao, Ph.D.

Division of Pharmacotherapy and Experimental Therapeutics, UNC School of Pharmacy

2318 Kerr Hall, UNC Eshelman School of Pharmacy

Chapel Hill, NC 27599-7569

E-mail: yanguang@unc.edu

Phone: +1-919-966-4040.

Supplementary Table 1. Non-linear mixed effect model parameter estimation.

Parameter	Definition	Unit	Estimate	Standard Error	Relative Standard Error (%)
Kg	Tumor progression rate	1/day	0.0033	5.04E-5	1.53
Kd	Tumor regression rate	1/day	0.0171	0.000152	0.887
F	Resistant cell fraction	1	0.121	0.00306	2.53
Ωkg	Inter-lesion variability of Kg (log-normal distribution)		1.24	0.0117	0.941
Ωkd	Inter-lesion variability of Kd (log-normal distribution)		0.732	0.00771	1.05
ΩF	Inter-lesion variability of F (logit distribution)		2.91	0.0248	0.852
b	Proportional error model		0.296	0.000904	0.305

Supplementary Table 2. Demographic information of head and neck squamous cell carcinomas patients in validation dataset.

**Variable**	N=393
Age, years (mean, sd)	57.8 (7.9)
Gender (n, %)	
Male	346 (88.0)
Female	47 (12.0)
Self-Reported Race (n, %)	
White/Caucasian	356 (90.6)
Asian	34 (8.7)
Hispanic/Latino	1 (0.3)
Other	2 (0.4)
Body Surface Area, m^2 (mean,sd)	1.8 (0.2)
Tumor Type (n, %)	
Hypopharynx	60 (15.3)
Larynx	124 (31.6)
Oral Cavity	104 (26.4)
Oropharynx	105 (26.7)
Prior Surgery (n, %)	
Yes	334 (85.0)
No	59 (15.0)
Prior Radiation (n, %)	
Yes	305 (77.6)
No	88 (22.4)
Treatment (n, %)	
Panitumumab plus Chemotherapy	198 (50.4)
Chemotherapy Alone	195 (49.6)
Response (n, %)	
Complete Response	11 (2.8)
Partial Response	122 (31.0)
Progressive Disease	42 (10.7)
Stable Disease	215 (54.7)
Not Evaluable	3 (0.8)
Metastatic organ number (n, %)	
1	122 (31.0)
2	145 (36.9)
3	87 (22.1)
>=4	39 (9.9)

Supplementary Table 3. Demographic and metastatic profiles of patients under k-means clusters.

**Variable**	Hetero-Organ (n=817)	Liver-First (n=930)	Lung-First (n=577)	Mono-Organ (n=1,345)	Other-First (n=639)
Age, years (mean, sd)	59.8 (11.1)	60.5 (10.7)	61.5 (10.5)	60.1 (10.6)	59.8 (11.2)
Gender (n, %)					
Male	476 (58.3)	562 (60.4)	351 (60.8)	793 (59.0)	356 (55.7)
Female	341 (41.7)	368 (39.6)	226 (39.2)	552 (41.0)	283 (44.3)
Self-Reported Race (n, %)					
White/Caucasian	754 (92.3)	844 (90.8)	521 (90.3)	1,205 (89.6)	559 (87.5)
Black/African American	13 (1.6)	19 (2.0)	12 (2.1)	41 (3.1)	19 (3.0)
Asian	17 (2.1)	31 (3.3)	19 (3.3)	49 (3.6)	26 (4.0)
Other	33 (4.0)	36 (3.9)	25 (4.3)	50 (3.7)	35 (5.5)
BMI, kg/m^2 (mean,sd)	26.1 (4.9)	26.1 (5.0)	26.5 (5.1)	26.2 (5.0)	26.4 (5.1)
Tumor Type (n, %)					
Colon	488 (59.7)	550 (59.1)	281 (48.7)	866 (64.4)	396 (62.0)
Rectal	265 (32.4)	303 (32.6)	256 (44.4)	356 (26.5)	179 (28.0)
Unspecified	64 (7.8)	77 (8.3)	40 (6.9)	123 (9.1)	64 (10.0)
Prior surgery (n, %)					
Yes	565 (69.2)	628 (67.5)	379 (65.7)	969 (72.0)	452 (70.7)
No	252 (30.8)	302 (32.5)	198 (34.3)	376 (28.0)	187 (29.3)
Prior radiation (n, %)					
Yes	588 (72.0)	705 (75.8)	390 (67.6)	1,160 (86.2)	502 (78.6)
No	89 (10.9)	88 (9.5)	101 (17.5)	91 (6.8)	76 (11.9)
Unknown	140 (17.1)	137 (14.7)	86 (14.9)	94 (7.0)	61 (9.5)
Metastatic organ number (n, %)					
1	0 (0)	0 (0)	53 (9.2)	482 (35.8)	18 (2.8)
2	0 (0)	305 (32.8)	185 (32.1)	444 (33.0)	225 (35.2)
3	176 (21.5)	315 (33.9)	188 (32.6)	270 (20.1)	197 (30.8)
≥4	641 (78.5)	310 (33.3)	151 (26.2)	149 (11.1)	199 (31.1)
KRAS status (n, %)					
Wild-Type	129 (15.8)	172 (18.5)	88 (15.3)	272 (20.2)	134 (21.0)
Mutant	106 (13.0)	142 (15.3)	75 (13.0)	190 (14.1)	80 (12.5)
Unknown	582 (71.2)	616 (66.2)	414 (71.8)	883 (65.7)	425 (66.5)
Has liver metastases at baseline (n, %)					
Yes	667 (81.6)	920 (98.9)	384 (66.6)	1,285 (95.5)	406 (63.5)
No	150 (18.4)	10 (1.1)	193 (33.4)	60 (4.5)	233 (36.5)
Baseline target tumor volume mm^3 (mean, sd)	243,091 (402,762)	240,895 (432,291)	169,061 (380,487)	192,247 (353,082)	122,878 (249,221)

Supplementary Table 4. The hyperparameters of the gradient boosting model to predict patient progression sequence.

Hyperparameters	Grid-search Range	Best Performed Model
Max_depth	3, 4, 5	3
Min_child_weight	1, 2, 5	2
Learning_rate	0.1	0.1
subsample	0.5	0.5
N_estimators	50, 100, 200	200
colsample_bytree	0.5	0.5

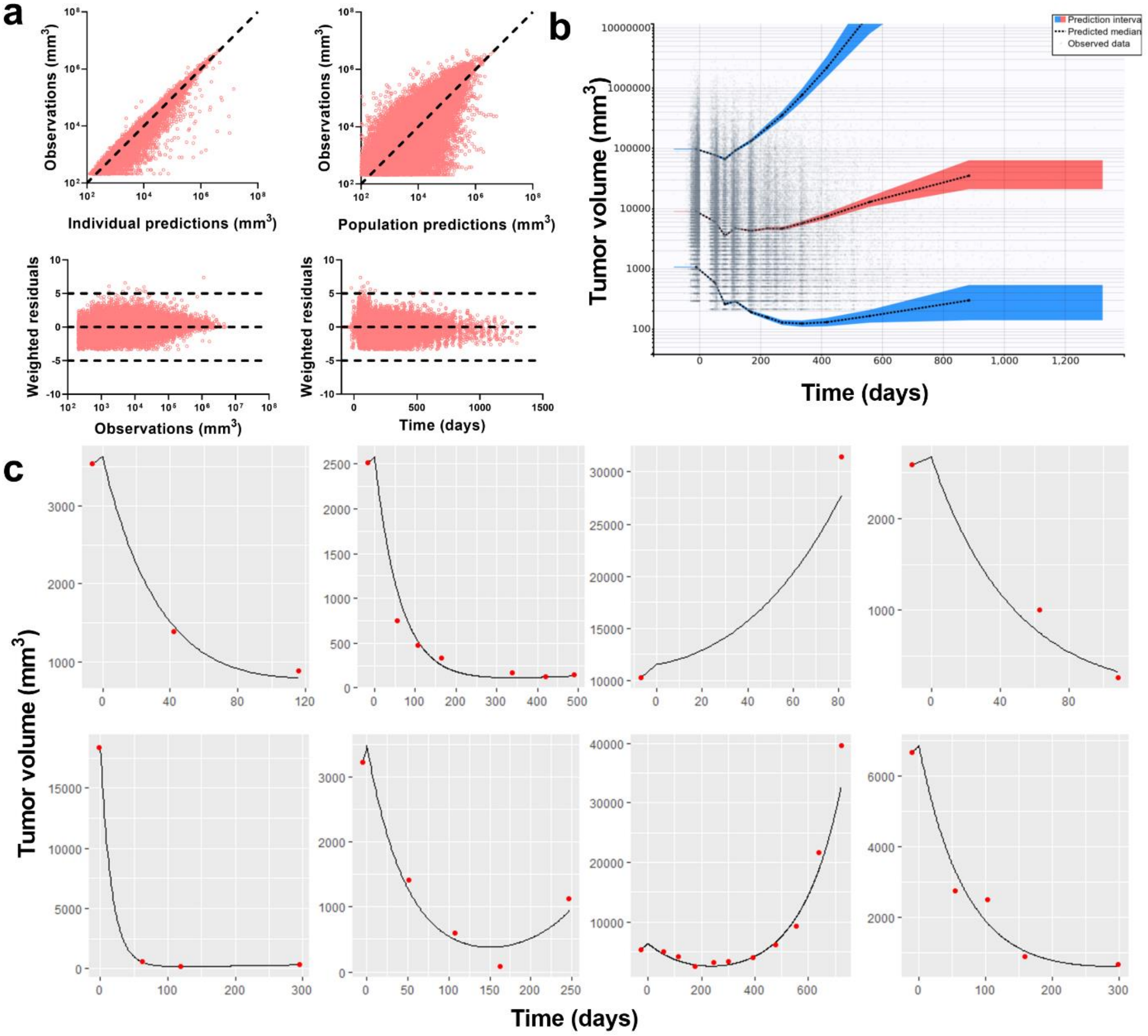

Supplementary Fig. 1 Tumor growth model has good model performance. a. Goodness-of-fit plots for tumor growth model. The upper panels are the observed tumor volume data plotted against individual model predictions (left) or population model predictions (right). The bottom panels are the weighted residuals plotted against observed tumor volume (left) or time (right). **b.** Visual predictive checks for tumor volume simulated from tumor growth model. The gray dots are the observations. The dashed lines describe the 10th, 50th, and 90th percentiles of model predictions and the bands are the model-simulated 90% confidence interval of the corresponding percentile. **c.** Eight representative lesions observed tumor volume (red circles) and the model predictions (black line) change with time. Source data are provided as a Source Data file.

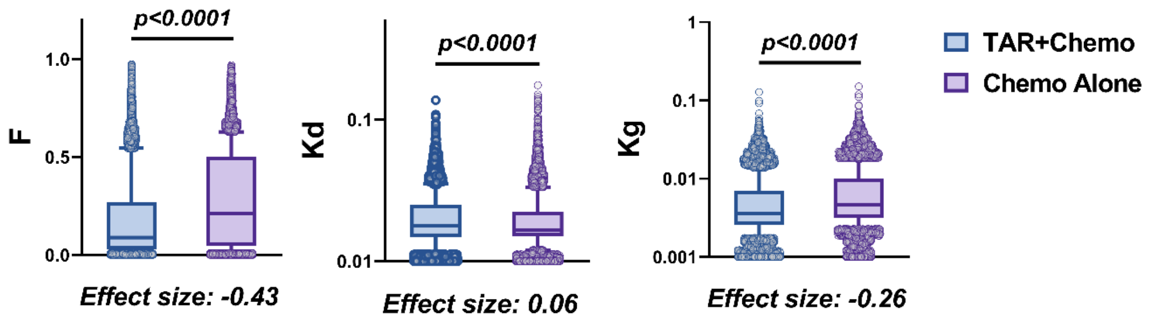

Supplementary Fig. 2 Comparisons of parameter *F*, *Kd*, *Kg* of lesions under antibody targeted therapies plus chemotherapy (TAR+Chemo, n=10,035) vs. chemotherapy alone (Chemo Alone, n=9,145). The box extends from the 25th to 75th percentiles and the line in the middle is plotted as the median. The whiskers are drawn down to the 10th percentile and up to the 90th percentile. Points below and above the whiskers are drawn as individual points. P-values are calculated by two-tailed Mann–Whitney test and the effect size are calculated by *Cohen'd*. Source data are provided as a Source Data file.

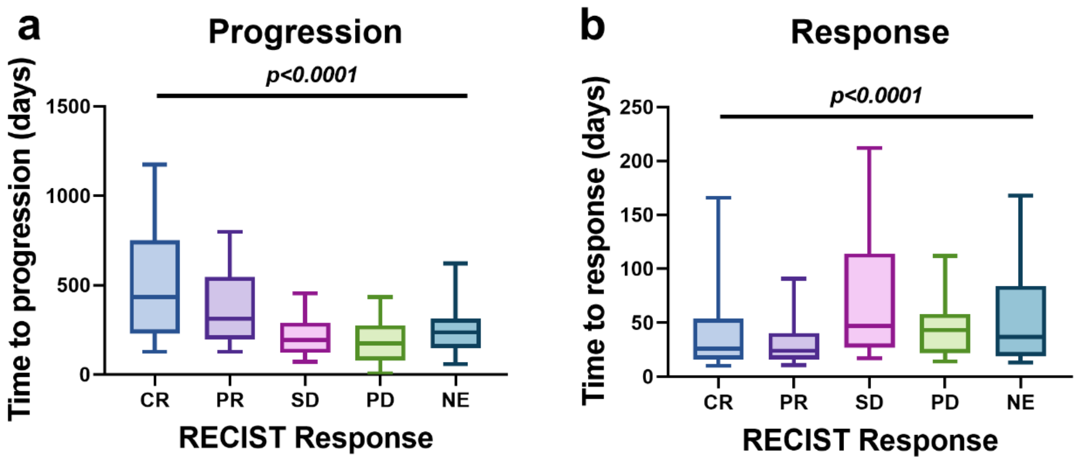

Supplementary Fig. 3. **Model predicted progression or response duration by patient response status determined by RECIST 1.0.** The response (**a**) or progression (**b**) times were predicted by tumor growth model and patient status of response were made per RECIST 1.0, including complete response (CR, n=118), partial response (PR, n=1,473), stable disease (SD, n=1,806), progressive disease (PD, n=781), and not evaluate (NE, n=130). The box extends from the 25th to 75th percentiles and the line in the middle is plotted as the median. The whiskers are drawn down to the 10th percentile and up to the 90th percentile. P-values were calculated by two-sided one-way ANOVA tests. Source data are provided as a Source Data file.

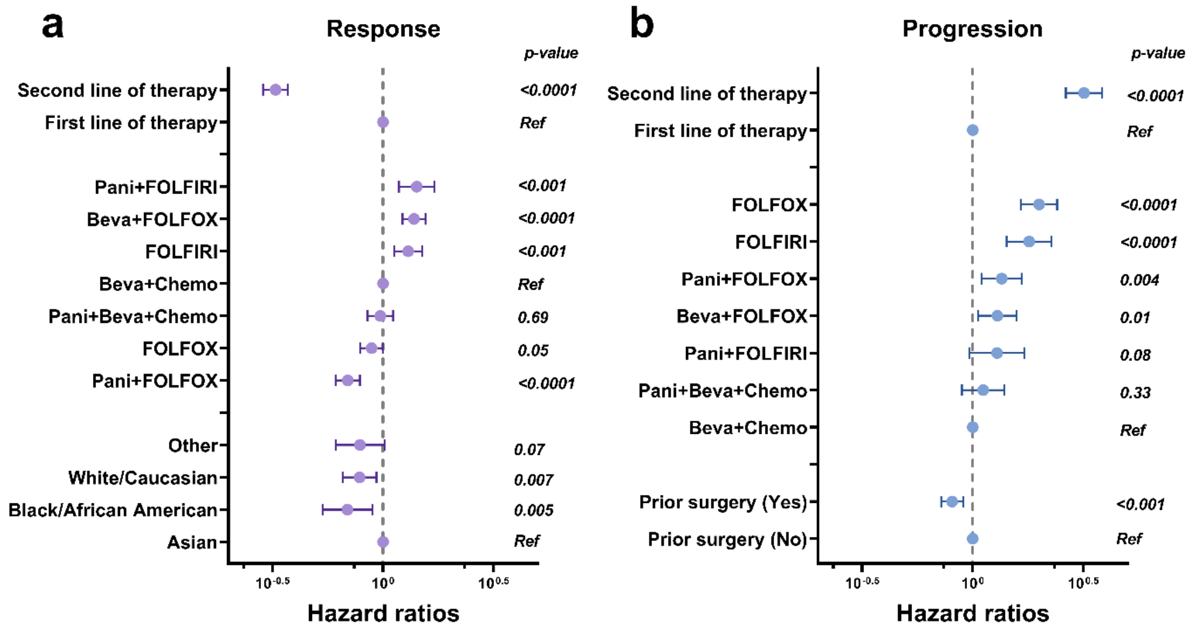

Supplementary Fig. 4. Covariate effects on lesion response and progression. **a** and **b** Data are presented as the hazard ratio estimates with 95% confidence interval of each potential covariate. P-values were calculated by two-sided likelihood ratio tests. Source data are provided as a Source Data file.

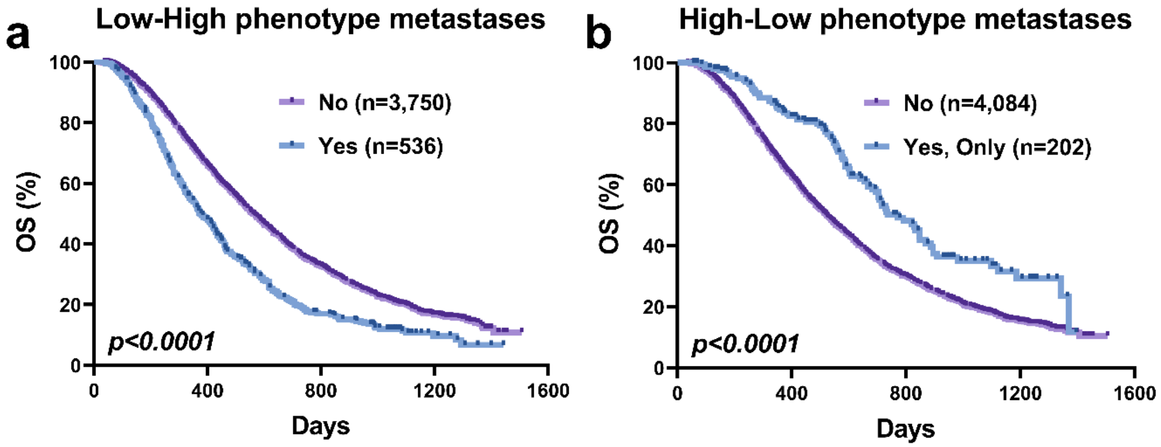

Supplementary Fig. 5 Metastasis Organ phenotypes are associated with patient prognosis. a. Patients without low-high phenotype metastases (genitourinary and reproductive system, adrenal, muscle/soft tissues, bone, brain/central nervous system) have significantly better survival than patients have low-high phenotype metastases. **b.** Patients only have metastases in high-low phenotype organs (lymph nodes, chest, spleen, lung) have significantly better survival than patients with other phenotypes. P-values were calculated by two-sided log-rank tests. Source data are provided as a Source Data file.

We also built Cox proportional models to patient overall survival and incorporated patient Low-High phenotype/High-Low phenotype and age, gender, race, treatment type, tumor type, prior surgery history, prior radiation history, BMI, *KRAS* type, metastatic organ number and metastatic lesion number as confounding factors. Patients with low-high phenotype metastases have significantly worse survival than patients without low-high phenotype metastases (Hazard Ratio = 1.21, $p<0.001$). Patients only have metastases in high-low phenotype organs have significantly better survival than those with other phenotypes (Hazard Ratio = 0.76, $p<0.05$).

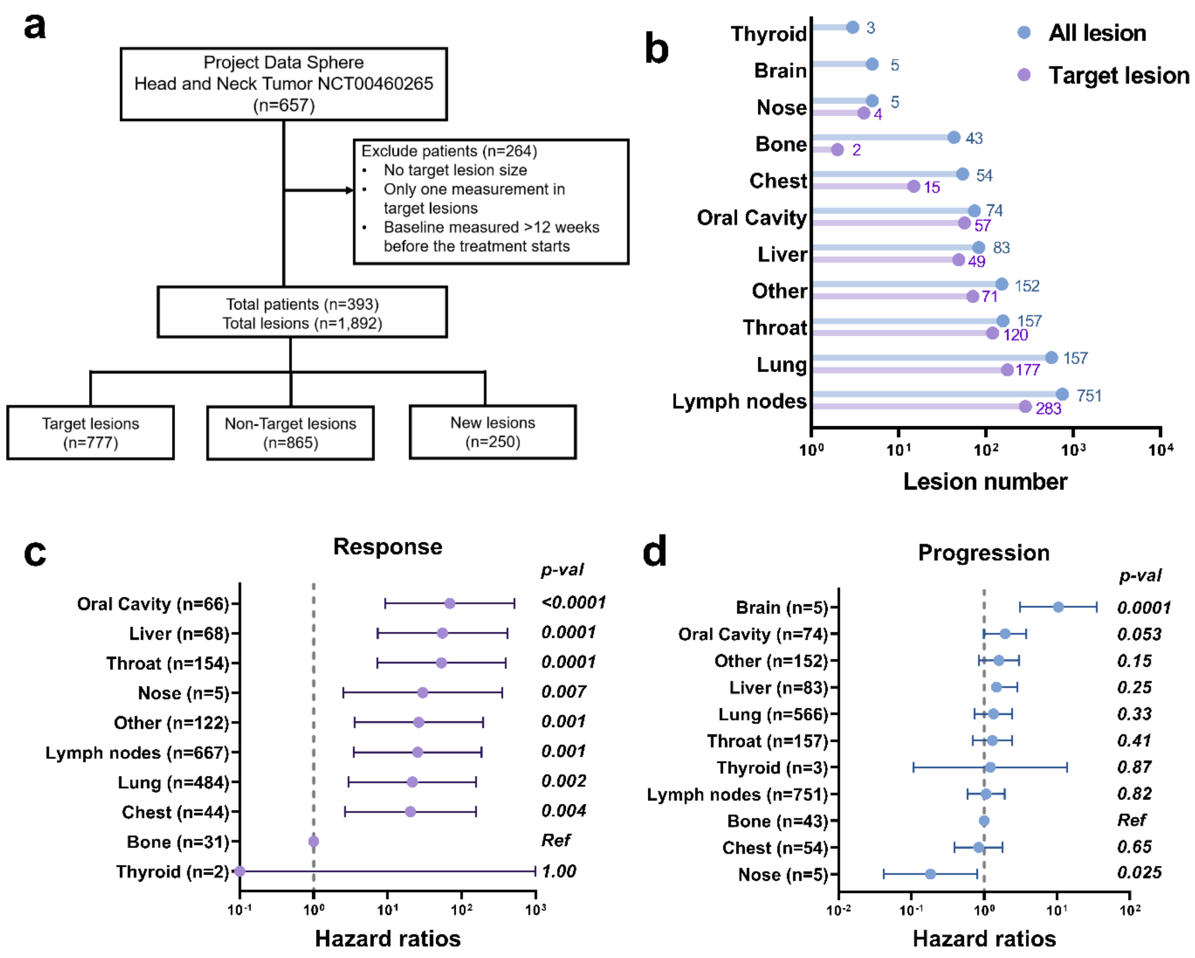

Supplementary Fig. 6 Validation of organ mapping in head and neck tumor. a. CONSORT diagram of patients and lesions included in data analysis. **b.** The number of all lesions and target lesions across organs. **c** and **d** Data are presented as the hazard ratio estimates with 95% confidence interval of lesion response and progression by organs. P-values were calculated by two-sided likelihood ratio tests. Source data are provided as a Source Data file.

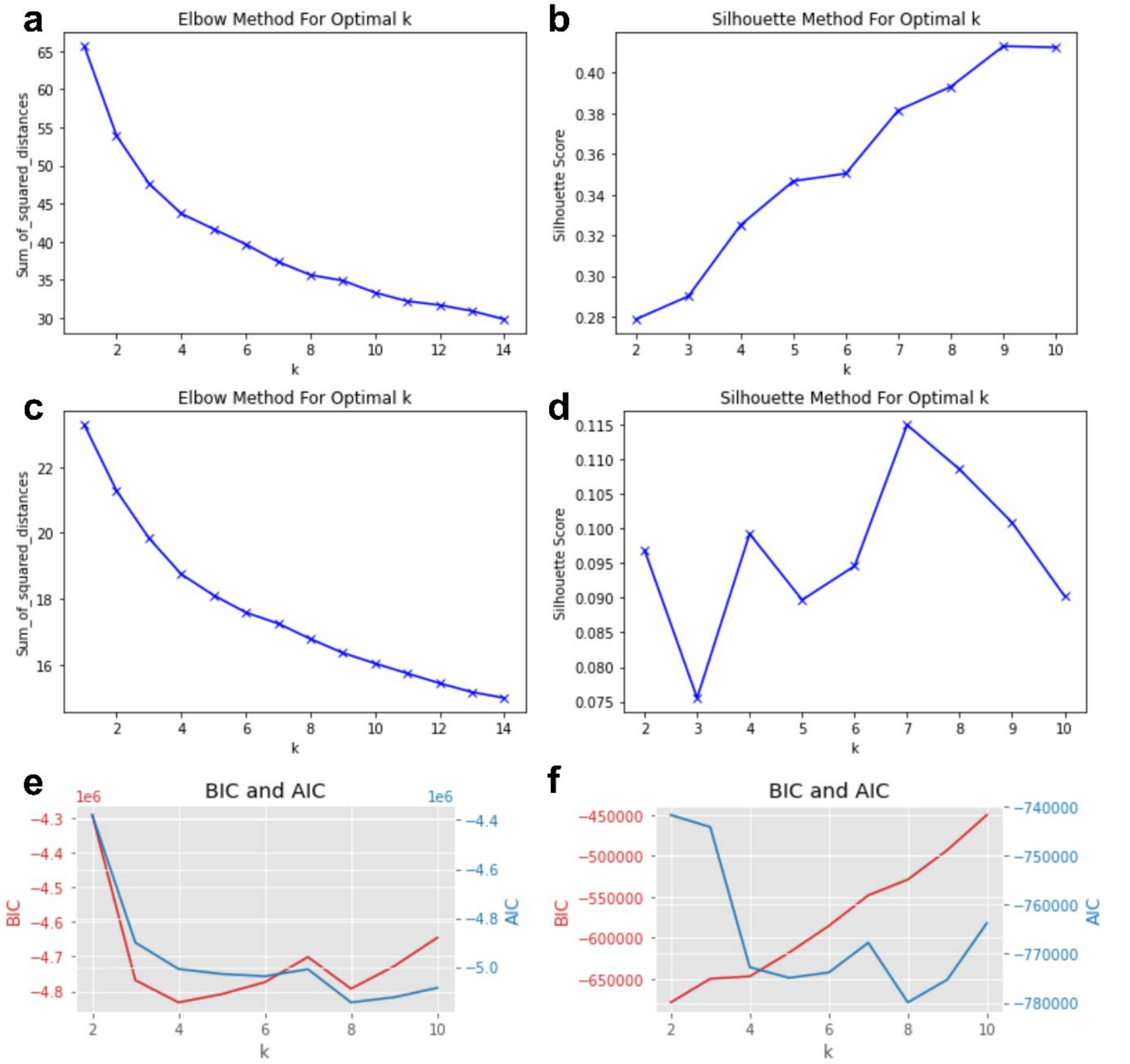

Supplementary Fig. 7 Determine optimal k for K-means. **a** and **b** Elbow method and Sihouette method were applied to find optimal k in progression patterns classification for all the patients. **c** and **d** Elbow method and Sihouette method were applied to find optimal k in progression patterns classification for Hetero-Organ patients. **e** and **f** Akaike information criterion (AIC) and Bayesian information criterion (BIC) across different k for all the patients (**e**) or Hetero-Organ patients (**f**). Source data are provided as a Source Data file.

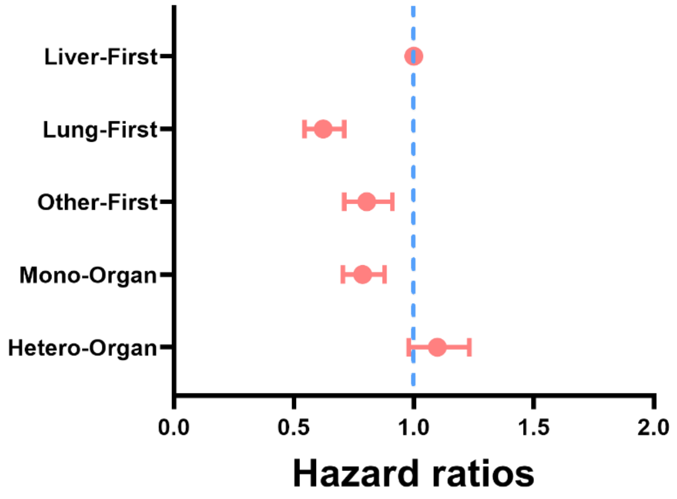

Supplementary Fig. 8 Patient progression pattern remains significant on overall survival in the presence of confounding factors. A Cox proportional hazard model predicting patient overall survival and incorporating patient progression clusters was built. Data are presented as the hazard ratio estimates with 95% confidence interval of patient cluster on overall survival. The following confounders were evaluated in the model: age, gender, race, treatment type, tumor type, prior surgery history, prior radiation history, BMI, *KRAS* type, metastatic organ number, and metastatic lesion number. Source data are provided as a Source Data file.

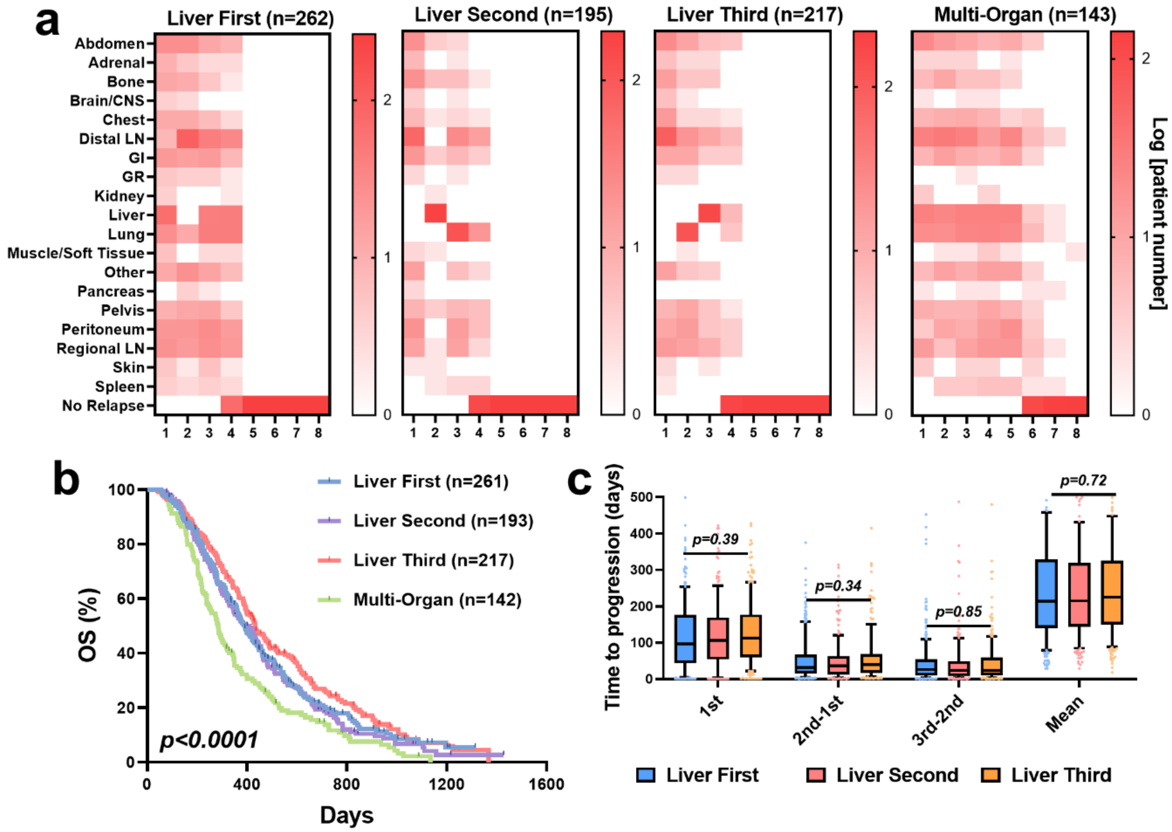

Supplementary Fig. 9 The progression sequence of liver metastases within the Hetero-Organ group is associated with patient survival. a. Hetero-Organ patients were clustered into four groups by liver progression sequence. **b.** Kaplan-Meier curves of patient overall survival by liver progression sequence. **c.** The boxplots of the first lesion progression time (1st), time between first and second progression (2nd-1st), time between second and third progression (3rd-2nd), time between third and fourth progression (4th-3rd), and the average progression time in Liver First (n=261), Liver Second (n=193), Liver Third (n=217), and Multi-Organ groups (n=142). The box extends from the 25th to 75th percentiles and the line in the middle is plotted as the median. The whiskers are drawn down to the 10th percentile and up to the 90th percentile. Points below and above the whiskers represent individual lesions. P-values in **b** was calculated by two-sided log-rank test and p-values in **c** were calculated by two-sided Dunn's multiple comparisons. Source data are provided as a Source Data file.

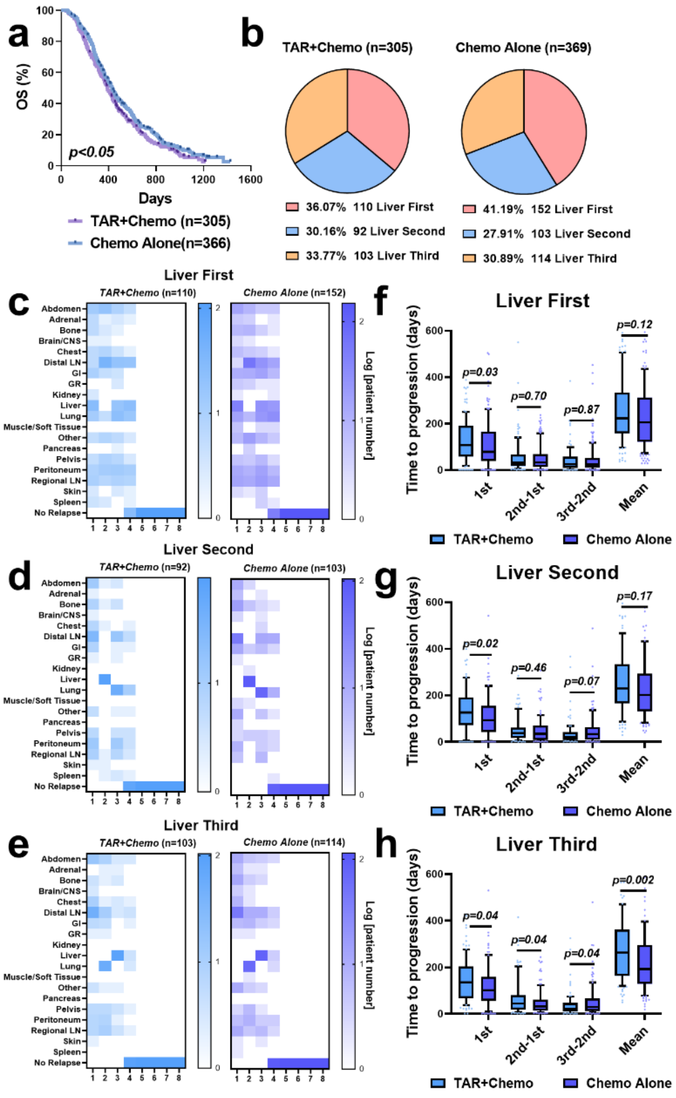

Supplementary Fig. 10 Targeted therapy has minimal effect on progression sequence. a. Three hetero-organ sub-groups in **Supplementary Fig. 6a** (Liver First, Liver Second, and Liver Third) patients overall survival stratified by treatments. **b.** Liver First, Liver Second, and Liver Third patient proportions by treatments. **c, d,** and **e** Patient progression sequences stratified by treatments. **f, g,** and **h** The box plots of the first lesion progression time (1st), time between first and second progression (2nd-1st), time between second and third progression (3rd-2nd), time between third and fourth progression (4th-3rd), and the average progression time by treatments of the groups in **c**, **d**, and **e**. N=110/n=92/n=103 patients from TAR+Chemo and n=152/n=103/n=114 patients from Chemo Alone were included in **f**, **g**, and **h.** The box extends from the 25th to 75th percentiles and the line in the middle is plotted as the median. The whiskers are drawn down to the 10th percentile and up to the 90th percentile. Points below and above the whiskers represent individual lesions. P-values in **f**, **g**, and **h** were calculated by two-sided Kruskal-Wallis tests. TAR+Chemo, antibody targeted therapies (bevacizumab or panitumumab) plus chemotherapy; Chemo Alone, chemotherapy alone. Source data are provided as a Source Data file.

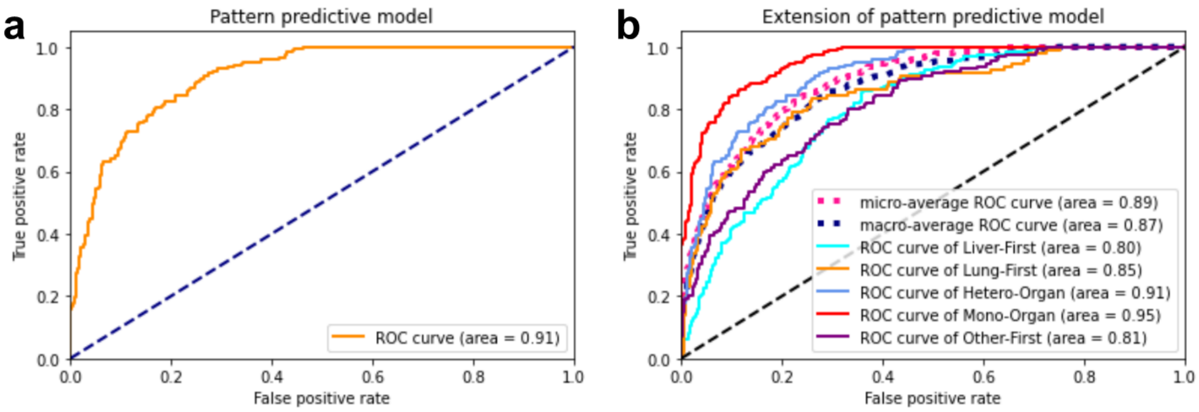

Supplementary Fig. 11 Progression sequence prediction model performance. a. The machine learning algorithm Gradient Boosting (XGBoost) model overall performance. **b.** Model performance stratified by sub-cluster. The results of the training group were: precision=0.76, accuracy =0.75, recall = 0.76. The results of the testing group were: precision = 0.59, accuracy = 0.60, recall = 0.58. Source data are provided as a Source Data file.
