# Supplementary material for: Mapping lesion-specific response and progression dynamics and inter-organ variability in metastatic colorectal cancer
Source: Nat Commun. 2023 Jan 26;14:417. doi: 10.1038/s41467-023-36121-y (PMC9876906; doi:10.1038/s41467-023-36121-y)
Supplement: Supplementary file 3 — Description to Additional Supplementary Information [file 41467_2023_36121_MOESM3_ESM.pdf]

Description of Additional Supplementary Files

Supplementary Data 1: A summary of clinical trials included in our analysis.
