# Supplementary material for: Mapping lesion-specific response and progression dynamics and inter-organ variability in metastatic colorectal cancer
Source: Nat Commun. 2023 Jan 26;14:417. doi: 10.1038/s41467-023-36121-y (PMC9876906; doi:10.1038/s41467-023-36121-y)
Supplement: Supplementary file 5 — Reporting Summary [file 41467_2023_36121_MOESM5_ESM.pdf]

nature portfolio

Corresponding author(s): Yanguang Cao

Last updated by author(s): 12/20/2022

Reporting Summary

Nature Portfolio wishes to improve the reproducibility of the work that we publish. This form provides structure for consistency and transparency in reporting. For further information on Nature Portfolio policies, see our Editorial Policies and the Editorial Policy Checklist.

Statistics

For all statistical analyses, confirm that the following items are present in the figure legend, table legend, main text, or Methods section.

n/a	Confirmed	
☐	☒	The exact sample size (*n*) for each experimental group/condition, given as a discrete number and unit of measurement
☐	☒	A statement on whether measurements were taken from distinct samples or whether the same sample was measured repeatedly
☐	☒	The statistical test(s) used AND whether they are one- or two-sided<br>*Only common tests should be described solely by name; describe more complex techniques in the Methods section.*
☐	☒	A description of all covariates tested
☐	☒	A description of any assumptions or corrections, such as tests of normality and adjustment for multiple comparisons
☐	☒	A full description of the statistical parameters including central tendency (e.g. means) or other basic estimates (e.g. regression coefficient) AND variation (e.g. standard deviation) or associated estimates of uncertainty (e.g. confidence intervals)
☐	☒	For null hypothesis testing, the test statistic (e.g. *F*, *t*, *r*) with confidence intervals, effect sizes, degrees of freedom and *P* value noted<br>*Give P values as exact values whenever suitable.*
☒	☐	For Bayesian analysis, information on the choice of priors and Markov chain Monte Carlo settings
☒	☐	For hierarchical and complex designs, identification of the appropriate level for tests and full reporting of outcomes
☐	☒	Estimates of effect sizes (e.g. Cohen's *d*, Pearson's *r*), indicating how they were calculated

Our web collection on statistics for biologists contains articles on many of the points above.

Software and code

Policy information about availability of computer code

Data collection	Clinical data was from Project Data Sphere website https://data.projectdatasphere.org/projectdatasphere/html/access. Access can be acquired on the Project Data Sphere website. The processed data generated in the study are provided in the Source Data file with this paper.
Data analysis	Clinical data were processed in in R-4.1.0 and RStudio 2022.07.1 dplyr package. Nonlinear mixed effect modeling was conducted using Monolix2020R1 Lixoft. Cox proportional models were built in R-4.1.0 and RStudio 2022.07.1 coxme package. k-means clustering algorithm was performed in Spyder (Python 3.8) in Anaconda using the SCIKIT-LEARN 1.0.2 software package. Gradient Boosting algorithm was built in Spyder (Python 3.8) in Anaconda using the SCIKIT-LEARN 1.0.2 software package. Data in machine learning analyses were processed using OneHotEncoder package softmax function. All the statistical tests were performed in GraphPad Prism 9. All software code is provided at Github: https://github.com/zhoujw14/Mapping-Metastasis.git

For manuscripts utilizing custom algorithms or software that are central to the research but not yet described in published literature, software must be made available to editors and reviewers. We strongly encourage code deposition in a community repository (e.g. GitHub). See the Nature Portfolio guidelines for submitting code & software for further information.

Data

Policy information about availability of data

All manuscripts must include a data availability statement. This statement should provide the following information, where applicable:

- Accession codes, unique identifiers, or web links for publicly available datasets
- A description of any restrictions on data availability
- For clinical datasets or third party data, please ensure that the statement adheres to our policy

The raw clinical data that support the findings of this study are available in the Project Data Sphere, https://data.projectdatasphere.org/projectdatasphere/html/access. Access can be acquired on the Project Data Sphere website. The processed data generated in the study are provided in the Source Data file with this paper.

Human research participants

Policy information about studies involving human research participants and Sex and Gender in Research.

Reporting on sex and gender	The gender number and ratio in the dataset was provided.
Population characteristics	All included data were metastatic colorectal cancer patients enrolled in Phase III trials. Details about patient demographic information were also provided in Table 1.
Recruitment	Trial inclusion/exclusion details were shared through Project Data Sphere, https://data.projectdatasphere.org/projectdatasphere/html/access. Patients were recruited by sponsors and investigators, no obvious selection bias present.
Ethics oversight	All trial number and ethic summary and their weblinks were provided. All study protocols were approved by institutional review boards at each participating center, including the clinical trial review boards from Amgen Inc., Pfizer Inc., Sanofi Inc., and AstraZeneca Inc.

Note that full information on the approval of the study protocol must also be provided in the manuscript.

Field-specific reporting

Please select the one below that is the best fit for your research. If you are not sure, read the appropriate sections before making your selection.

☒ Life sciences ☐ Behavioural & social sciences ☐ Ecological, evolutionary & environmental sciences

For a reference copy of the document with all sections, see nature.com/documents/nr-reporting-summary-flat.pdf

Life sciences study design

All studies must disclose on these points even when the disclosure is negative.

Sample size	no sample size calculation was performed in this study. patient number in each enrolled trial was provided. The sample size chosen based on data availability and pre-set criteria (methods). The sample size in our pooled studies is 4308, which is greater than most Phase III clinical trials and is sufficient for our analyses.
Data exclusions	detailed data exclusion criteria was provided in Figure 1 and method. Patients under one of the following conditions were excluded: (1) no target lesion longitudinal measurements; (2) baseline tumor size measured more than 12 weeks before the treatment. We excluded patients without longitudinal tumor size measurements since we need the tumor size data to build tumor growth dynamic model. Patients whose baseline tumor size measured more than 12 weeks before treatment might experience other therapeutic interventions during this period.
Replication	the results could be reproduced with the provided data sources and the shared model code.
Randomization	Our study is a retrospective analysis based on the randomized studies, but no randomization was involved in our analyses.
Blinding	the investigators were not blinded to allocation during data analyses and outcome assessments since it is a retrospective analysis.

Reporting for specific materials, systems and methods

We require information from authors about some types of materials, experimental systems and methods used in many studies. Here, indicate whether each material, system or method listed is relevant to your study. If you are not sure if a list item applies to your research, read the appropriate section before selecting a response.

Materials & experimental systems

n/a	Involved in the study
☒	☐ Antibodies
☒	☐ Eukaryotic cell lines
☒	☐ Palaeontology and archaeology
☒	☐ Animals and other organisms
☐	☒ Clinical data
☒	☐ Dual use research of concern

Methods

n/a	Involved in the study
☒	☐ ChIP-seq
☒	☐ Flow cytometry
☒	☐ MRI-based neuroimaging

Clinical data

Policy information about clinical studies

All manuscripts should comply with the ICMJE guidelines for publication of clinical research and a completed CONSORT checklist must be included with all submissions.

Clinical trial registration

NCT00384176; NCT00457691; NCT00561470; NCT00305188; NCT115765; NCT00364013

Study protocol

All study protocols were available in Project Data Sphere, https://data.projectdatasphere.org/projectdatasphere/html/access

Data collection

The raw clinical data that support the findings of this study are available in the Project Data Sphere, https://data.projectdatasphere.org/projectdatasphere/html/access. Access can be acquired on the Project Data Sphere website. The study recruitment time range from July 2002 to August 2015. The processed data generated in the study are provided in the Source Data file with this paper.

Outcomes

The trial outcomes were all different, and the summary of study outcomes for each clinical trial were accessible in Project Data Sphere https://data.projectdatasphere.org/projectdatasphere/html/access. The most common primary endpoint is patient progression-free survival (defined as the start of therapies until RECIST-defined progression or death) , and most common second outcome is patient overall survival (defined as the start of therapies until patient death). Both endpoints were collected in our study and were analyzed.
